# A registry based analysis of the patient reported outcome after surgery for trapeziometacarpal joint osteoarthritis

**DOI:** 10.1186/s12891-020-3045-7

**Published:** 2020-02-01

**Authors:** Maria Wilcke, Martin Roginski, Mikael Åström, Marianne Arner

**Affiliations:** 1 Karolinska Institute, Department of Clinical Science and Education, Södersjukhuset, Stockholm, Sweden; 2 Department of Hand surgery, Södersjukhuset, Sjukhusbacken 10, 11883 Stockholm, Sweden; 3 Region Skåne, Department of Data Analytics and Register Centrum, Lund, Sweden

**Keywords:** Trapeziometacarpal joint, CMC1, Thumb base, Osteoarthritis, Thumb, Patient reported outcome measures, Quality registry, Hand surgery

## Abstract

**Background:**

The aim of the study was to evaluate patient reported outcome measures (PROM) before and after trapeziectomy with or without ligament reconstruction and tendon interposition for trapeziometacarpal joint arthritis with special focus on possible differences due to gender, age and surgical method.

**Methods:**

Data from the Swedish quality registry for hand surgery (HAKIR) was analyzed preoperatively, 3 months and 1 year postoperatively for 1850 patients (mean age 63 years, 79% women).

**Results:**

One year postoperatively, mean pain at rest was reduced from 50 to 12 of maximum 100. However, pain on load and weakness had not abated to the same extent (mean 30 and 34 of 100, respectively). The mean improvement in PROM did not differ between age groups or gender. The result was similar after trapeziectomy with ligament reconstruction and tendon interposition (86% of the patients) and simple trapeziectomy but few patients were operated with the latter method.

**Conclusion:**

Pain on load and weakness remains to some extent 1 year after surgery for trapeziometacarpal joint arthritis. The result is similar after trapeziectomy with or without ligament reconstruction and tendon interposition and the same improvement can be expected after surgery regardless of age and gender.

## Background

The trapeziometacarpal joint (TMJ) is a common site of osteoarthritis, especially in elderly women [1, 2]. When non-operative treatment is insufficient, trapeziectomy with ligament reconstruction and tendon interposition (LRTI) still remains the dominant surgical method, although the literature has not shown any advantages compared to simple trapeziectomy [3–7]. The effect of surgical interventions for TMJ osteoarthritis in terms of patient reported outcome measures (PROM) has been assessed with for example the Michigan Hand Outcomes Questionnaire (MHQ) [8], the Patient Evaluation Measure (PEM) [9], and most commonly the Disability of the Arm, Shoulder and Hand (DASH) questionnaire [2, 10]. PROMs before and after surgery for TMJ osteoarthritis have previously not been studied in large patient materials.

The first national healthcare quality registry for hand surgery was started in Sweden in 2010 [11]. The registry is named HAKIR and includes all operations performed at the seven specialist departments of hand surgery in Sweden, as well as two private units. PROM questionnaires are issued to all operated patients before, as well as 3 and 12 months after surgery and include the QuickDASH [12] and an eight-item questionnaire (HQ-8) with seven questions rating perceived symptoms in the operated hand (pain on load, pain on motion without load, pain at rest, stiffness, weakness, numbness and cold sensitivity) and one question about the ability to perform activities of daily living (ADL) (Additional file 1). The responses are used as single items and are not calculated into a total score. The HQ-8 uses a nine-level Likert scale in 11 point increments ranging from 0 (no problem) to 100 (worst problem imaginable) and has been shown to have good psychometric properties in a yet unpublished study by Carlsson et al. (2019). The QuickDASH has shown similar precision as the full length DASH in upper extremity disorders [13].

The primary aim of this study was to evaluate what effect trapeziectomy with or without LRTI have on HQ-8 and QuickDASH scores in a large cohort of patients with TMJ osteoarthritis, with special focus on possible gender and age differences. The secondary aim was to investigate the practice of simple trapeziectomy vs. LRTI and potential differences in results in terms of HQ-8 and QuickDASH scores.

## Methods

**Fig. 1:**
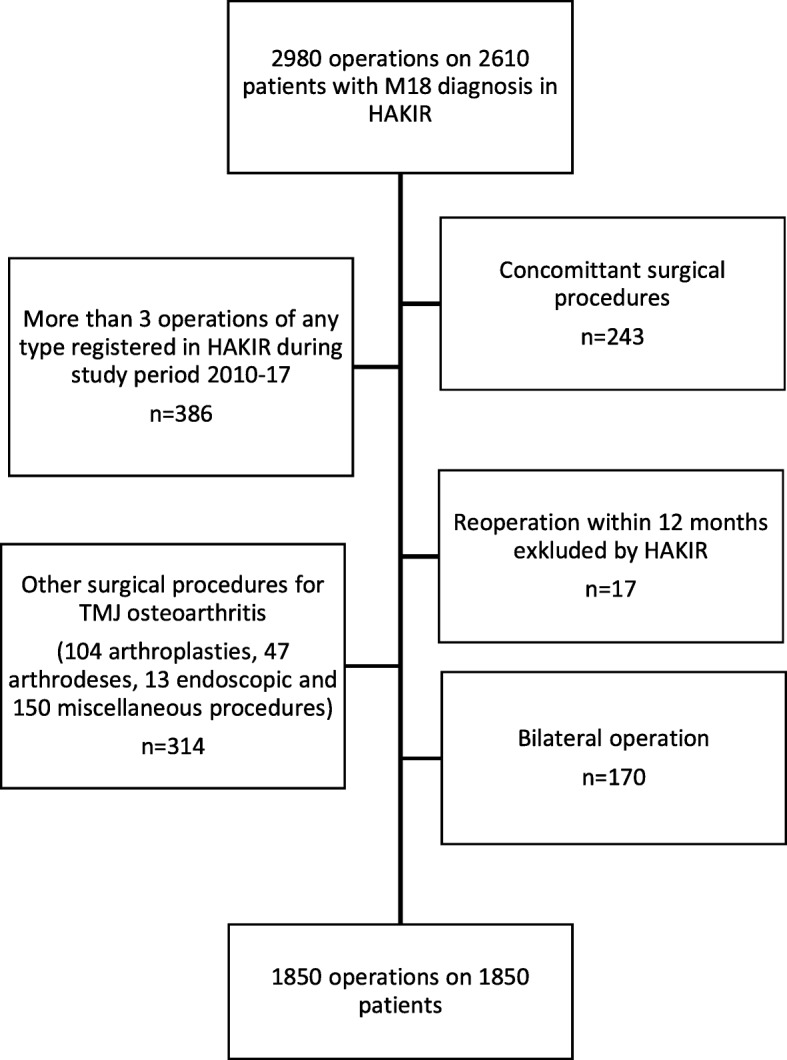
Flow charts of the patients

Registry data from HAKIR for all patients operated for TMJ arthritis (ICD10 code M18) from the start of the registry on February 1, 2010 to September 2, 2017 was analyzed. In total, 2980 operations were registered in 2610 patients. Only patients operated with trapeziectomy with or without LRTI were included. Hence, patients operated with other methods were excluded (Fig. 1). A concomitant major surgical procedure was also an exclusion criterion. However, patients undergoing minor concomitant surgeries such as trigger finger, carpal tunnel release, ganglion excision or fusion of the metacarpophalangeal joint of the thumb were not excluded from the analysis. In patients operated on bilaterally (on separate occasions), the second operation was excluded. Patients who had more than three recorded surgical interventions in the hand during the period were excluded to reduce the risk for other conditions affecting the PROM. The final study population included 1850 operations in 1850 patients operated unilaterally with trapeziectomy with or without LRTI. The type of LRTI could not be distinguished in the registered data.

In HAKIR, patient questionnaires are issued either as a web-form or in a paper version that is posted to the patient. Patients who have been re-operated within 1 year, are below 16 years of age or persons that for cognitive reasons are unable to complete a questionnaire are excluded from the postoperative surveys. Response rates in HAKIR have varied from 43% in 2014 to 51% in 2017 (www.HAKIR.se). 46% (*n* = 852) of the 1850 patients had completed the questionnaire preoperatively, 42% (*n* = 774) at 3 months post-operatively and 37% (*n* = 683) 1 year after the operation.

The HQ-8 questionnaire items concerning pain on load, pain on motion without load, pain at rest, stiffness, weakness and ability to perform activities of daily living (ADL) as well as total score for the QuickDASH were evaluated.

PROM are presented as mean (SD) for all responses preoperatively, 3 months and 1 year postoperatively. The mean individual improvement in PROM from preoperatively to 1 year postoperatively is presented for patients with responses at both occasions. The patient material was dichotomized in two age groups at the mean age. Student’s unpaired t-test (2-sided) was used for comparisons of PROM values between age groups, gender and surgical treatment. Missing data is not compensated for when all responses are presented and compared. Significance level was set at 0.05.

An analysis of non-responders regarding age, gender and type of operation was made by comparing the patients that had answered the pain on load question preoperatively but not at 1 year to those that had answered this item at both occasions. Student’s unpaired t-test (2-sided) and Chi-square test were used for these comparisons.

## Results

Baseline data is presented in Table 1. The analysis on responders (337) vs. non-responders (515) showed the same distribution of gender and operation methods. The mean age of the non-responders was lower than the responders (62 vs. 64 years, *p* < 0.01).

**Table 1:** Baseline data

	All	Women	Men
Number	1850	1464	386
Mean age (years)	63	63	64
Median age /years)	63	62	64
Range (years)	28–93	32–39	28–89
Simple trapeziectomy	250	189	61
Trapeziectomy + LRTI	1600	1275	325

**Fig. 2:**
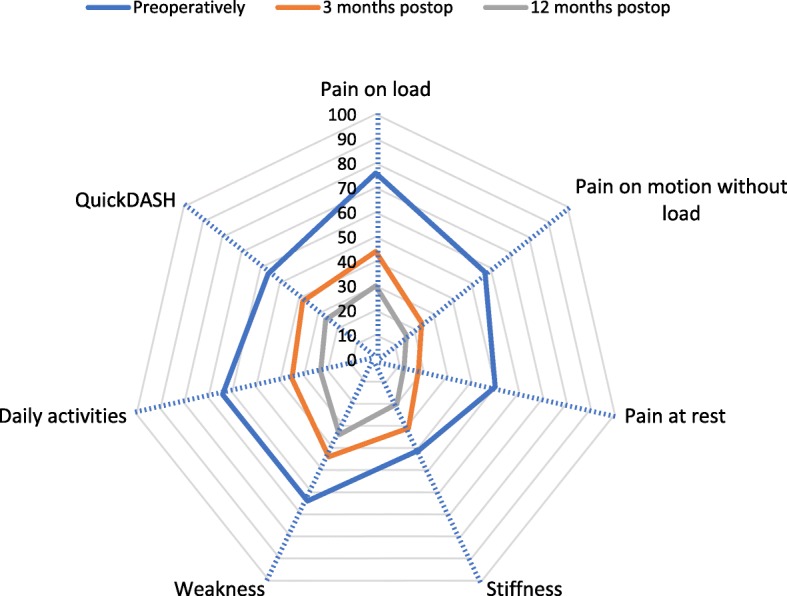
The change of mean HQ-8 and Quick DASH scores (0–100) for all 1850 patients

The PROM responses for all patients are shown in Fig. 2. Table 2 presents the mean PROM values, number of responses at the different times, and the mean individual change.

**Table 2:** The mean PROM values

PROM	Preop PROM mean (SD)	No. of responses preop	3 months PROM mean (SD)	No. of responses 3 months	1 year PROM mean (SD)	No. of responses 1 year	Mean individual change (SD) preop - 1 year	No. of responses preop - 1 year
Pain on load	76 (17)	852	44 (25)	770	30 (26)	675	−44 (29)	337
Pain on motion without load	57 (22)	851	24 (22)	774	16 (22)	679	−40 (27)	338
Pain at rest	50 (25)	847	18 (22)	772	12 (19)	681	−36 (28)	336
Stiffness	41 (28)	837	31 (24)	770	20 (23)	683	−18 (33)	337
Weakness	64 (24)	847	44 (26)	770	34 (27)	678	−30 (31)	333
Daily activities	64 (23)	850	35 (26)	770	24 (25)	677	−39 (32)	336
QuickDASH	56 (16)	843	38 (20)	758	26 (20)	667	−31 (19)	330

Prom values preoperatively, 3 months and 1 year postoperatively and the mean individual change. PROM values range from 0 (no problem) to 100 (worst problem imaginable)

Patients 63 years or younger (*n* = 1015) reported significantly worse scores for pain (on load, on motion without load and at rest) and ADL preoperatively and significantly worse scores for all parameters except pain at rest and QuickDASH postoperatively compared to patients 64 years or older (Table 3). However, the differences were small and the mean individual reported improvement did not differ between the age groups.

**Table 3:** PROM values according to age groups

	Age group (years)	Preoperative mean (SD)	3 months mean (SD)	1 year mean (SD)	Individual change Preop − 1 year mean (SD)*
Pain at load	28–63	78 (16)	49 (25)	34 (27)	−44 (29)
	64–93	73 (18)	39 (25)	26 (25)	−44 (30)
		*p* < 0.001	*p* < 0.001	*p* < 0.001	*p* = 0.83
Pain on motion without load	28–63	60 (21)	26 (23)	18 (22)	−42 (27)
	64–93	54 (23)	21 (21)	13 (21)	−39 (27)
		*p* < 0.001	*p* < 0.001	*p* = 0.005	*p* = 0.36
Pain at rest	28–63	53 (24)	21 (24)	13 (20)	−39 (27)
	64–93	46 (26)	15 (19)	11 (17)	−33 (28)
		*p* < 0.001	*p* < 0.001	*p* = 0.14	*p* = 0.06
Stiffness	28–63	42 (27)	35 (25)	22 (23)	−18 (35)
	64–93	39 (28)	26 (23)	18 (23)	−19 (32)
		*p* = 0.15	*p* < 0.001	*p* = 0.03	*p* = 0.72
Weakness	28–63	65 (22)	48 (25)	37 (26)	−28 (30)
	64–93	63 (25)	39 (25)	32 (27)	−32 (33)
		*p* = 0.37	*p* < 0.001	*p* = 0.01	*p* = 0.32
ADL	28–63	67 (23)	39 (26)	26 (26)	−40 (31)
	64–93	61 (24)	31 (24)	21 (24)	−38 (32)
		*p* = 0.001	*p* < 0.001	*p* = 0.02	*p* = 0.65
Quick DASH	28–63	57 (16)	41 (20)	28 (20)	−31 (18)
	64–93	56 (16)	34 (20)	25 (20)	−31 (19)
		*p* = 0.09	*p* < 0.001	*p* = 0.06	*p* = 0.96

The mean PROM values preoperatively, 3 months and 1 year postoperatively and the mean individual change according to age groups. PROM values range from 0 (no problem) to 100 (worst problem imaginable) * The mean individual improvement in PROM from preoperatively to 1 year postoperatively is presented for patients with responses at both occasion

The mean scores for the majority of symptoms were significantly higher preoperatively for women (Table 4). Postoperatively, women reported significantly higher scores concerning pain at rest, weakness, problems in ADL and QuickDASH. As for the age groups, the differences were small and the mean individual reported improvement was similar between genders.

**Table 4:** PROM values according to gender

	Gender	Preoperative mean (SD)	3 months mean (SD)	1 year mean (SD)	Individual change Preop − 1 1 year mean (SD)
Pain at load	women	76 (17)	44 (25)	31 (26)	-43 (39) *n* = 280
	men	73 (17)	43 (26)	26 (26)	-49 (25) *n* = 57
		*p* = 0.06	*p* = 0.55	*p* = 0.08	*p* = 0.11
Pain on motion without load	women	59 (22)	24 (22)	16 (21)	-41 (28) *n* = 281
	men	52 (24)	21 (22)	15 (22)	-38 (23) *n* = 57
		*p* < 0.001	*p* = 0.10	*p* = 0.60	*p* = 0.51
Pain at rest	women	52 (24)	19 (22)	12 (19)	-37 (29) *n* = 279
	men	43 (25)	15 (19)	9 (18)	-33 (23) *n* = 57
		*p* < 0.001	*p* = 0.10	*p* = 0.05	*p* = 0.29
Stiffness	women	42 (28)	31 (24)	20 (23)	-18 (33) *n* = 281
	men	38 (27)	29 (24)	18 (23)	-19 (32) *n* = 56
		*p* = 0.21	*p* = 0.31	*p* = 0.42	*p* = 0.84
Weakness	women	66 (23)	44 (26)	36 (27)	-29 (31) *n* = 276
	men	58 (27)	42 (25)	28 (25)	-35 (32) *n* = 57
		*p* < 0.001	*p* = 0.46	*p* = 0.003	*p* = 0.19
ADL	women	65 (23)	36 (25)	24 (25)	-38 (32) *n* = 280
	men	59 (23)	31 (27)	19 (24)	-44 (28) *n* = 56
		*p* = 0.002	*p* = 0.04	*p* = 0.05	*p* = 0.18
QuickDASH	women	58 (16)	39 (20)	28 (21)	−31 (20) *n* = 273
	men	48 (16)	34 (22)	19 (18)	−31 (18) *n* = 57
		*p* < 0.001	*p* = 0.01	*p* < 0.001	*p* = 0,99

The mean PROM values preoperatively, 3 months and 1 year postoperatively and the mean individual change according to gender. PROM values range from 0 (no problem) to 100 (worst problem imaginable)

At 3 months, stiffness, ADL and QuickDASH were significantly better in the simple trapeziectomy group but the differences were small and there were no differences in mean PROM scores between simple trapeziectomy and LRTI after 1 year (Table 5). The mean individual improvement according to operation type was not analyzed since there were only complete responses both preoperatively and after 1 year in 39 patients with simple trapeziectomy (*n* = 298 patients with LRTI).

**Table 5:** PROM values according to surgical method

PROM	Operation	Preoperatively		3 months		1 year	
		n=	Mean (SD)	n=	Mean (SD)	n=	Mean (SD)
Pain on load	Trapeziectomy	102	74 (17)	102	40 (25)	87	30 (25)
	LRTI	750	76 (17)	668	45 (25)	588	30 (26)
			*p* = 0,38		*p* = 0,07		*p* = 0,99
Pain on motion without load	Trapeziectomy	102	53 (25)	103	21 (21)	87	16 (22)
	LRTI	749	58 (22)	671	24 (22)	592	16 (22)
			*p* = 0,07		*p* = 0,18		*p* = 0,97
Pain at rest	Trapeziectomy	100	47 (25)	103	16 (21)	87	11 (19)
	LRTI	747	50 (25)	669	18 (22)	594	12 (19)
			*p* = 0,29		*p* = 0,37		*p* = 0,78
Stiffness	Trapeziectomy	101	38 (29)	103	26 (23)	87	19 (23)
	LRTI	736	41 (27)	667	37 (24)	596	21 (23)
			*p* = 0,32		*p* = 0,02		*p* = O,65
Weakness	Trapeziectomy	103	64 (25)	102	41 (24)	86	35 (25)
	LRTI	744	64 (24)	668	44 (26)	592	34 (27)
			*p* = 0,85		*p* = 0,30		*p* = 0,79
Daily activities	Trapeziectomy	102	62 (22)	101	30 (23)	86	23 (25)
	LRTI	748	64 (23)	669	36 (26)	591	23 (25)
			*p* = 0,38		*p* = 0,03		*p* = 0,92
QuickDASH	Trapeziectomy	102	55 (17)	102	33 (19)	87	24 (19)
	LRTI	741	56 (16)	656	39 (20)	580	26 (20)
			*p* = 0,49		*p* = 0,01		*p* = 0,37

The mean PROM values preoperatively, 3 months and 1 year postoperatively after simple trapeziectomy versus ligament reconstruction and tendon interposition (LRTI). PROM values range from 0 (no problem) to 100 (worst problem imaginable)

## Discussion

One year after surgery for TMJ joint osteoarthritis, most patients had experienced a major reduction of pain at rest from a mean score of 50 to 12 and the mean QuickDASH score was reduced by more than half. However, patients should be informed that a complete resolution of pain on load and weakness not is to be expected 1 year after surgery (mean score 30 and 34, respectively). Younger patients and women reported slightly worse PROM both before and after surgery but the mean individual improvement in PROM did not differ between age groups or gender.

Our data may be used for preoperative patient information about the expected outcome after surgery. Further, the presented data can be useful as reference values for power calculations in the design of studies comparing interventions for TMJ joint osteoarthritis and also applied as benchmark values to which the results of observational studies can be compared.

The registry data confirmed that trapeziectomy and LRTI still is the prevailing surgical method in Sweden despite reports that LRTI has shown no advantage over simple trapeziectomy but rather a higher risk for complications [2, 4, 14]. Reports from the United States [6, 15] demonstrate the same tendency that clinical practice does not follow available evidence. The mean PROM values for all patients favored simple trapeziectomy at 3 months postoperatively regarding stiffness, ADL and QuickDASH but the differences were probably too small to be relevant. Results were similar 1 year postoperatively which is in line with former studies [2, 4, 14]. We could not make a well-founded comparison of the individual improvement after simple trapeziectomy versus LTRI due to low response rates and the fact that so few of the former method was performed. Thus, we cannot make any recommendations regarding surgical method based on this study. It was not possible to distinguish different type of LRTI in the data so LRTI:s were by necessity grouped as one category which might not be optimal. However, there is no solid evidence that results differ after various LRTI [3].

The strong point of this study is the large sample. This is valuable in analysis of subjective variables such as PROM that inherently have a large individual variance and might be affected by other factors such as depression or other upper-extremity comorbidities [16, 17]. To reduce the uncertainty due to individual variance, we made paired analyses of the individual improvement in PROM. Due to low response rates and the fact that many patients did not respond at all three occasions, the samples were markedly reduced in the paired analyses and this affected in particular the comparison between surgical methods.

A problem with the large sample is that small differences in PROM that might not be clinically important, may reach statistical significance. The minimum clinically important difference (MCID) for QuickDASH was determined by Franchignoni et al. [18] to16 points. MCID for HQ-8 items have not yet been described. In general, MCID tend to be 0,5 SD [19]. We found (statistically) significantly worse PROM scores in younger patients and in women both before and after surgery, but none of the differences in the HQ-8 item scores were close to 0,5 SD. QuickDASH was significantly higher in women at all times but the difference was 10 points at most. The differences could be attributable to higher functional demands in younger patients and differing ADL and life-style habits, anatomical variability or pain perception between men and women but the differences may as well represent normal variation. Since the effect of the operation (i.e. improvement of PROM) did not differ between age groups or gender, we do not interpret our results as younger patients and female having an inferior result after operation.

Registry studies enable inclusion of many more patients than randomized controlled trials (RCTs), which often compare relatively few patients treated under strictly controlled conditions. Moreover, registry studies report the “real life” situation, including all types of patients, treated at different centers and operated by many different surgeons. For many hand conditions, RCTs is almost impossible to perform due to small populations and we believe that registry studies will be increasingly important in the field of hand surgery. On the other hand, potential confounders cannot not be controlled for in registry studies which might induce e.g. selection bias regarding surgical method.

A limitation of this study is that the sample did not include re-operated patients and we have no information on complication rates. Patients who sustained postoperative complications probably would have affected the patient reported outcomes negatively. Further, 1 year is a relatively short follow-up time and the result may change with time. Yeoman et al. [20] report a mean quick-DASH of 40 after 3,5–17 years after simple trapeziectomy which is considerably higher than in this sample (QuickDASH 26). The poor response rate is a major limitation of the HAKIR and thus of this study. The HQ-8 questionnaire is presently issued mainly by e-mail, as a web-survey. There is a risk that e-mails end up in spam filters or that patients are not motivated to respond*.* One reminder to answer the questionnaire is send by a sms. There is a risk that the older population answers web-surveys to a lesser extent. However, the mean age of the non-responders was actually lower than responders. For all ages, there is a risk for survey fatigue so simple questionnaires with few questions are probably favorable for a better response rate and we believe that the HQ-8 fulfill this request. More information to patients about the registry and its purpose could be an incentive. Improved response rate is a crucial improvement needed for the HAKIR to reach its full potential to compare hand surgical interventions.

## Conclusion

Good patient-reported outcomes, especially concerning pain at rest, can be expected 1 year after trapeziectomy with or without LRTI in most patients. Remaining symptoms may be pain on load and weakness. Further data needs to be collected for valid comparison between simple trapeziectomy and LRTI.

## Supplementary information

**Additional file 1.** HQ-8 questionnaire.

## Data Availability

The datasets used and/or analyzed during the current study are available from the corresponding author on reasonable request.

## Abbreviations

ADL: Activities of daily living
DASH: Disability of the Arm, Shoulder and Hand
HAKIR: The Swedish quality registry for hand surgery
HQ-8: The HAKIR 8-item questionnaire
LRTI: Ligament reconstruction and tendon interposition
PROM: Patient reported outcome measures
TMJ: Trapeziometacarpal joint
