# Supplementary material for: A registry based analysis of the patient reported outcome after surgery for trapeziometacarpal joint osteoarthritis
Source: BMC Musculoskelet Disord. 2020 Feb 1;21:63. doi: 10.1186/s12891-020-3045-7 (PMC6995059; doi:10.1186/s12891-020-3045-7)
Supplement: Supplementary file 1 — Additional file 1. HQ-8 questionnaire. [file 12891_2020_3045_MOESM1_ESM.pdf]

HAKIR
HANDKIRURGISKT
KVALITETSREGISTER

Date of birth (social security no) (yyyymmdd-nnnn): ☐☐☐☐☐☐☐☐–☐☐☐☐

Patient questionnaire HQ-8 (arm/hand)

Date (yyyy-mm-dd) ☐☐☐☐-☐☐-☐☐

I am (please indicate your writing hand): ☐ Left handed ☐ Right handed ☐ Ambidextrous

Arm/hand that was operated on: ☐ Left ☐ Right

This questionnaire reports on problems that you have had this past week in the hand/arm that was operated on. Please tick the alternative that best corresponds to any of your problems.

1. Pain on load

	0	10	20	30	40	50	60	70	80	90	100	
No problems	☐	☐	☐	☐	☐	☐	☐	☐	☐	☐	☐	Worst problems imaginable

2. Pain on motion without load

	0	10	20	30	40	50	60	70	80	90	100	
No problems	☐	☐	☐	☐	☐	☐	☐	☐	☐	☐	☐	Worst problems imaginable

3. Pain at rest

	0	10	20	30	40	50	60	70	80	90	100	
No problems	☐	☐	☐	☐	☐	☐	☐	☐	☐	☐	☐	Worst problems imaginable

4. Stiffness

	0	10	20	30	40	50	60	70	80	90	100	
No problems	☐	☐	☐	☐	☐	☐	☐	☐	☐	☐	☐	Worst problems imaginable

5. Weakness

	0	10	20	30	40	50	60	70	80	90	100	
No problems	☐	☐	☐	☐	☐	☐	☐	☐	☐	☐	☐	Worst problems imaginable

6. Numbness / tingling in fingers

	0	10	20	30	40	50	60	70	80	90	100	
No problems	☐	☐	☐	☐	☐	☐	☐	☐	☐	☐	☐	Worst problems imaginable

7. Cold Sensitivity (discomfort on exposure to cold)

	0	10	20	30	40	50	60	70	80	90	100	
No problems	☐	☐	☐	☐	☐	☐	☐	☐	☐	☐	☐	Worst problems imaginable

8. Ability to perform daily activities

	0	10	20	30	40	50	60	70	80	90	100	
No problems	☐	☐	☐	☐	☐	☐	☐	☐	☐	☐	☐	Worst problems imaginable
